# Phylogenetics of tick-borne encephalitis virus in endemic foci in the upper Rhine region in France and Germany

**DOI:** 10.1371/journal.pone.0204790

**Published:** 2018-10-18

**Authors:** Malena Bestehorn, Sebastian Weigold, Winfried V. Kern, Lidia Chitimia-Dobler, Ute Mackenstedt, Gerhard Dobler, Johannes P. Borde

**Affiliations:** 1 Parasitology Unit, University of Hohenheim, D-Stuttgart, Germany; 2 Division of Infectious Diseases, Department of Medicine II, University of Freiburg Medical Center and Faculty of Medicine, Freiburg i.Br., Germany; 3 Bundeswehr Institute of Microbiology, German Center of Infection Research (DZIF) partner site Munich, Neuherbergstraße 11, München, Germany; 4 Praxis Dr. J. Borde / Gesundheitszentrum Oberkirch, Am Marktplatz 8, Oberkirch, Germany; Ain Shams University (ASU), EGYPT

## Abstract

**Objective:**

Tick-borne encephalitis (TBE) caused by the tick-borne encephalitis virus (TBEV) is the most important tick-borne arboviral disease in Europe and Asia. The Upper Rhine Valley is thought to be the very western border of TBEV distribution in Europe. The aim of our study was to identify natural foci and isolate TBEV from ticks, to determine the prevalence of TBEV in local tick populations and to study the phylogenetic relatedness of circulating TBEV strains in this region.

**Material and methods:**

Ticks were collected between 2016, 2017 and 2018 by flagging. TBEV was isolated from collected ticks and phylogenetic analyses were performed. Minimal infection rates (MIR) of the collected ticks were calculated.

**Results:**

At 12 sampling sites, a total of 4,064 Ixodes ticks were collected in 2016 and 2017 –(and one single collection 2018). 953 male, 856 female adult ticks and 2,255 nymphs were identified. The MIR rates were 0,17% (1/595) for Schiltach (Germany) and 0,11% (1/944) for Foret de la Robertsau (France), respectively. Overall, the three newly described TBEV strains, isolated in the years 2016 and 2017 from the Upper Rhine Valley have no close phylogenetic relation and show a genetic relationship with strains from eastern Europe. The 2018 TBEV strain from Aubachstrasse (Germany), however, is closely related to the TBEV found in Schiltach (Germany).

**Conclusion:**

In conclusion, we demonstrate, to our knowledge for the first time, the phylogenetic relations of the newly isolated TBEV strains on both sides of the upper Rhine river.

## Introduction

Tick-borne encephalitis (TBE) is the most important tick-borne arboviral disease in Europe and Asia resulting in more than 10.000 cases every year [1,2]. The causative agent is the Tick-borne encephalitis virus (TBEV), which is mainly transmitted to humans by hard ticks. The main vector in Central Europe is the hard tick *Ixodes ricinus*. In 2016 TBEV was the first time isolated from *Dermacentor reticulatus* ticks in the federal state of Saxonia in Germany (pers. communication G. Dobler and M. Pfeffer), similar findings are reported from other Eastern European countries before [3–5]. Rodents (e.g. *Apodemus* spp. *and Myodes* spp.) serve as the main reservoir hosts for TBEV in the natural cycle between ticks and vertebrate hosts. Alimentary transmission of TBEV by unpasteurized dairy products plays an important role in eastern Europe, where foodborne disease outbreaks have been reported in the last decades [6–8]. The first documented alimentary TBEV infection after consumption of raw goat-milk products in Germany occured in the district of Reutlingen in Germany in 2016 [9].

TBEV is a member of the family Flaviviridae, genus *Flavivirus* with a single-stranded positive-sense RNA of approximately 11 kb. TBEV can by divided in three subtypes–the European (TBEV-EU), the Siberian (TBEV-Sib) and the Far-Eastern subtype (TBEV-FE). Infections with the different TBEV subtypes result in significant differences of the clinical picture, morbidity and mortality [1,2]. In Germany, exclusively the TBE-EU subtype has been isolated from rodent and tick samples so far. Human isolates are rarely available because of the short TBEV viremia in the very early symptomatic phase of the disease and the absence of virus in cerebrospinal fluid during the CNS symptomatic.

In Germany, TBE became a notifiable disease in 2001. Systematic public health data from 2001 until today show an annual number of reported TBE cases ranging between 200 and 450 (https://survstat.rki.de/Content/Query/Create.aspx). The majority of TBE cases are found in the federal states of Bavaria and Baden-Wuerttemberg. The local incidence shows significant variations–however, in some districts e.g. the Ortenaukreis (federal state of Baden-Wuerttemberg) it is estimated at about >10/100.000. This region is adjacent to the French region of Alsace. Alsace is the only French region where since 1968 stable endemic TBEV foci have been identified based on autochthonous TBEV infections [10,11]. Overall, about 170 human TBE cases had been reported in France from 1968 until 2016 –almost exclusively from the Alsace department. The Upper Rhine Valley and the Netherlands are thought to be the very western limits of TBEV-EU distribution in Europe and therefore focus of intensive virological and epidemiological studies.

There are however only few data available on the prevalence of TBEV in ticks in this region. Since the 1970s no data about the phylogenetic characteristics of the local TBEV-EU strains have been reported. Hence, the phylogenetic relationship of TBEV strains from the Upper Rhine Valley and the Alsatian TBEV strains as well as their distribution and possible expansion of distribution have not been studied. The aim of our study is to identify natural foci and isolate TBEV from ticks, to determine the prevalence of TBEV in the local tick populations and to study the genetic variability of local TBEV isolates and the phylogenetic relatedness of the circulating TBEV-EU strains at the western borders of the TBEV endemic regions. Local datasets from 2012, investigating the phylogenetic links between Bavarian and Czech TBEV foci, indicated a non-continuous spreading, with long distance migration of TBEV [12]. Of particular interest is the spatiotemporal comparison with historical isolates from Alsace [13,14], which might provide insight into the genetic stability of TBEV of endemic foci over time, the relevance of short and long distance distribution of TBEV strains and the potential role of rivers as barrier for the distribution of TBEV.

## Material and methods

### Samples and sampling sites

Ticks were collected 2016, 2017 and one single collection in 2018 by flagging at the following sites on the basis of non-systemic data of patients, anamnestic hints and publications. GPS/Glonass data are provided in S1 Table. Ticks were morphologically identified at the Bundeswehr Institute of Microbiology (TBEV national reference laboratory, Munich) at the species level and pooled according to developmental stage and sex (10 nymphs and 5 adult female or male ticks). We confirm that the field studies did not involve endangered or protected species. There were no specific permissions required for these locations/activities in the field. There are no restrictions regarding tick flagging in the described regions/districts. In case flagging was done on private ground—owner were asked for permission.

### TBEV Cultivation and Isolation

TBEV positive tick homogenates were cultivated in cell culture. Therefore A549 ATCC® CCL-185™ cells were infected with 300 μL of a 1:10 dilution of the homogenate, as published in the literature before [15]. After one hour of inoculation the cells were washed with PBS and cultivation media was added (MEM, 1xNEAA, 2%FCS). The cultivation was harvested after three to four days. The success of the viral culture was checked using a real-time RT-PCR [16]. All discussed TBEV strains in this paper were cultivated and isolated.

### Total RNA extraction, RT-PCR and Sequencing of PCR products

The ticks were pooled in Lysing Matrix A tubes (MP Biomedicals, Eschwege, Germany) with 1 mL media (Minimal Essential Medium, 3% Fetal Calf Serum, 10 fold antibiotics and antimycotics) and subsequently homogenized using the MP Tissue Lyser (MP, Eschwege, Germany). Total nucleic acid extraction was performed using MagNA Pure LC Total Nucleic Acid Isolation Kit (Roche, Mannheim, Germany) and the automated isolation and purification instrument MagNa Pure LC (Roche, Mannheim, Germany) according to the manufacturer’s instructions. Isolated nucleic acid was eluated in 50μl volume and stored at -80.0°C until further analysis. The samples were screened for TBEV RNA using RT-PCR [16–18]. Furthermore, PCR-positive results were confirmed by amplification of the viral E gene [15]. The E gene amplicons were purified and isolated using the QIAquick Gel Extraction and QIAquick PCR Purification Kits (Qiagen, Hamburg, Germany); E gene products and specific primers (Table 1) were send out for external sequencing services provided by GATC Biotech (Konstanz, Germany). DNA sequence analysis, generation of phylogenetic data and trees were analyzed by the Bundeswehr Institute of Microbiology.

**Table 1:** Sequencing primers.

*primer*	*primer-function*	*sequence (5' -> 3')*	*length [nt]*	genome position*
*TBE-885*	E-gene forward primer	GGT TAC CGT TGT GTG GTT GAC C	22	885–906
*TBE-c1648*	E-gene sequencing-primer	GCA GAG CCA GAT CAT TGA ACC	21	1628–1648
*TBE-2571a*	E-gene reverse primer 1	CTC CGG GTA GTA **G**GC ATA ATT G	22	2550–2571
*TBE-2571b*	E-gene reverse primer 2	CTC CGG GTA GTA **T**GC ATA ATT G	22	2550–2571

* reference genome TBEV strain Neudoerfl.

### DNA sequence analysis

All sequence data were processed using the program Geneious 9.1.5. A de novo assembly was performed using the three chromatograms obtained from GATC for each positive sample. Nucleotides with an estimated error higher than one percent were trimmed. Subsequently the sequences were cut to 1488 bp, the exact length of the envelope gene sequence. A ClustalW Alignment with several other e genes from selected isolates was performed and a phylogenetic tree was generated using the PhyML algorithm [19]. We used further the RAxML version 8 tool for advanced and more reliable phylogenetic analysis [20].

## Results

At 12 sampling sites a total of 4,064 *Ixodes ricinus* ticks were collected in 2016 and 2017. 953 male, 856 female adult ticks and 2,255 nymphs were identified. Four tick-pools from 3 potentially endemic sites were subsequently tested positive for TBEV RNA (Table 2). Particularly at the sampling sites Schiltach (G) and Foret de la Robertsau (F) there has been anecdotal evidence of severe human TBE cases in the last 20 years.

**Table 2:** Samples and sampling sites, 2016–2017 (G denotes for Germany, F denotes for France).

*sampling site*	*date*	*adult male ticks*	*adult female ticks*	*nymphs*	*total number (n)*	*TBEV positive*
*Burgerwald (G)*	May 16	22	23	40	85	
*Foret Neuhof (F)*	May 16	17	12	113	142	
*Renchen (G)*	May 16	90	65	36	191	
*Aubachstrasse (G)*	May 16	81	65	243	389	1 adult male
*Obersasbach (G)*	May 16	7	13	6	26	
*Foret de la Robertsau (F)*	May 16	25	22	94	141	
*Foret de la Robertsau (F)*	May 16	40	49	408	497	
*Sasbachwalden (G)*	May 16	28	22	11	61	
*Vogelweg (G)*	May 16	9	5	52	66	
*Guebviller (F)*	Jun 16	18	28	233	279	
*Schiltach (G)*	Mar 17	58	47	62	167	
*Aubachstrasse (G)*	Mar 17	20	13	56	89	1 adult female
*Antogast (G)*	Mar 17	10	12	4	26	
*Oppenau (G)*	Apr 17	72	69	18	159	
*Schiltach (G)*	Apr 17	114	81	400	595	1 nymph
*Sasbachwalden (G)*	May 17	7	7	5	19	
*Oppenau (G)*	May 17	20	22	5	47	
*Foret de la Robertsau (F)*	Jun 17	310	295	339	944	1 nymph
*Guebviller (F)*	Okt 17	5	6	130	141	
*total number (n)*		953	856	2255	4064	

### Minimal infection rates (MIR)

Minimal infection rates of the collected ticks (2016–2017) were calculated (Table 3). The latest collection 2018 at Aubachstrasse (G) was not included in the MIR calculations. Overall, at all three sites, where samples tested positive for TBEV, the MIR was lower than 1,0%. In detail, the sampling site Aubachstrasse (G) was flagged in 2016 and 2017, consequently resulting in positive TBEV samples in both years. The (overall) total MIR was 0,47% (2/478). In 2016 there was one TBEV positive pool of male ticks identified and in 2017 one pool of female ticks. TBEV positive pools containing nymphs were found in samples from Schiltach (G) and Foret de la Robertsau (F) in 2017. The MIR rates were 0,17% (1/595) for Schiltach (G) and 0,11% (1/944) for Foret de la Robertsau (F) respectively.

**Table 3:** Sampling sites and minimal infection rates (MIR) of TBEV positive ticks, 2016–2017. (G denotes for Germany, F denotes for France).

*sampling site*	*total MIR*	*male MIR*	*female MIR*	*nymph MIR*
*Aubachstrasse (G)*	0,42% (2/478)	0,99% (1/101)	1,28% (1/78)	0/299
*Schiltach (G)*	0,17% (1/595)	0/114	0/81	0,25% (1/400)
*Foret de la Robertsau (F)*	0,11% (1/944)	0/310	0/295	0,29% (1/339)

### Phyleogeographic analysis

**Fig 1:**
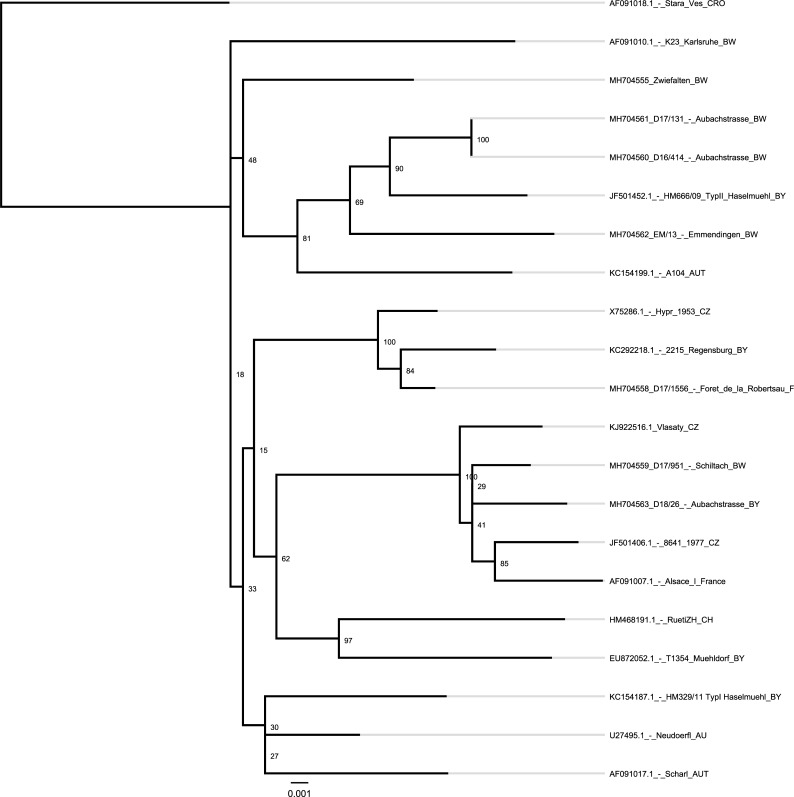
Phylogenetic data. Phylogenetic analysis of all isolated TBEV samples 2016–2018 using the RAxML tool version 8.

The newly isolated TBEV envelope gene sequences were compared with 16 selected regional, national and international TBEV strains from the NCBI database and the database of the German national reference laboratory (Fig 1)–including the reference strain Neudoerfl. A total of 9 E gene sequences from southwestern Germany and Alsace were available for phylogenetic analysis. Five E gene sequences are from newly isolated TBEV from 3 sampling sites (Aubachstrasse (G), Schiltach (G) and Foret de la Robertsau (F)). 2 TBEV E gene sequences from sequentional flagging expeditions in 2016 and 2017 were identical.

There were no significant differences using the PhyML algorithm (S1 Fig) or the RAxML tool (Fig 1) for the generation of the phylogenetic trees.

The isolated TBEV strains from Auchbachstrasse (G) were similar in 2016 and 2017. The phylogenetic results show a strong relationship to other TBEV strains from Emmendingen district in Baden-Wuerttemberg. The TBEV strain from Aubachstrasse (G) is clustering together with TBEV samples isolated from ticks in eastern Bavaria and Austria. However, the isolate from the first flagging expedition in 2018 showed a very different TBEV E gene sequence, which is clustering with the strain from Schiltach (G) and the historical TBEV isolate from Alsace. The TBEV strain isolated in 2016 and 2017 has not yet been detected this year at the sampling site in Auchbachstrasse (G).

The isolated and sequenced TBEV strain from Foret de la Robertsau (F) is related to circulating TBEV isolates from eastern Bavaria and the Czech Republic. The historical TBEV strain from Alsace from 1971 [13] can be associated with the newly found TBEV strain from Schiltach (G) and strains from the northeastern part of the Czech Republic. In the French department Alsace, there are at least two independent TBEV strains circulating–taken the historical and the newly identified sample together. Overall, the three newly described TBEV strains, isolated in the years 2016 and 2017 from the Upper Rhine Valley have no close phylogenetic relation and might rather form independent clades. These strains show a close genetic relationship with strains from eastern Europe underscoring an east to west dynamic of the TBEV distribution (S2 Fig). The 2018 TBEV strain from Aubachstrasse (G), however, is closely related to the TBEV found in Schiltach (G).

## Discussion

The aim of our study was the identification of TBE natural foci and the isolation and phylogenetic characterization of the circulating TBEV strains in the Upper Rhine Valley region. We followed a strategy to identify the place of infection of human cases and then sample there directly for TBEV, as the sampling in randomly selected areas, even in endemic areas proved to be of low success [21]. Our strategy proofed successful as we could detect two natural foci (Schiltach, Germany; Foret de la Robertsau, France) where humans acquired infections in previous years. Furthermore, we identified a randomly selected area as TBE natural focus (Aubachstrasse, Germany), where so far no human TBE case had been associated. As the identification of low- or non-pathogenic TBEV strains in nature has been proven, pathogenetic analyses have to be conducted to prove the pathogenetic potential of this TBEV strain [22].

The MIRs found in the three natural foci were very low and confirm that even in natural foci the prevalence of TBE virus in the tick populations is low. The rates ranged from 0.11% to 0.47%. In Schiltach and Foret de la Robertsau TBEV was detected only in nymphs. The MIRs were 0.11% and 0.17%. In Aubachstrasse the TBEV was detected in adult ticks and the overall MIR was 0.47% using the data from 2016 and 2017, which is about three to four times higher than in nymphs. These data are in agreement with findings from other studies in Central Europe. The results confirm that even directly in TBE natural foci only few ticks are infected with TBEV and hundreds of ticks have to be tested to confirm or disconfirm a TBEV natural focus [23].

The genetic analysis of the isolated and identified TBEV strains from three locations in Germany and France showed that there are at least three different TBEV strains detectable, indicating a significant regional genetic diversity. In the French department Alsace two TBEV strains have been identified and sequenced in the last 50 years. Surprisingly the ancient TBEV strain from Alsace is genetically linked to the newly identified strain from Schiltach (G) and the 2018 TBEV isolate from Aubachstrasse (G), which are approximately 50 km away from the historic sampling site. These findings indicate that this strain is still circulating in the region and again proves the high stability of TBEV as also seen in Finland. The detection of a closely related TBEV strains in the German focus Schiltach shows that the strain expanded its geographical range. It is interesting that the virus appears on both sides of the Rhine river. It had been recently shown that the Main river seems to form a barrier for the distribution of *Borrelia burgforferi* s.l., a bacterium, which is also transmitted by *Ixodes ricinus*, similar to TBEV [24]. However, there is now conclusive model of explanation of this finding at the moment.

On a broader scale (S2 Fig), all newly detected and sequenced TBEV strains and the historical TBEV sequence from Alsace are related to TBEV strains in eastern Bavaria, the Czech Republic and Austria. A very similar molecular phyleogeographic East to West pattern was described before in the Czech Bavarian border region for central Europe [12]. Several landscape, biogenic and anthropogenic features, which influence TBEV spread could be discussed in this context, e.g. along river systems or by circumpassing mountain ranges. Even the cold war iron curtain seemed to influence the TBEV spread by separating Bohemian-Bavarian red deer (*Cervus elaphus*) populations, which serve as tick-hosts and have large resident ranges [12]. Local virus spreading may facilitated by wild boar (*Sus scrofa*), which are able to cross the Rhine river. Wild boar is the most important bisulcate host of *I*. *ricinus* beside roe deer (*Capreolus capreolus*) in this region. The natural habitat of wild boar is larger and this species is able to migrate longer distances up to 40 km, whereas roe deer have a smaller radius of migration and habitat. There are several studies which indicate a link between wild boar population and local and regional spreading patterns of TBE [25–27]. In the Netherlands TBEV positive serum samples were detected from wild boars long before the first human TBE case occurred in 2016 [28]. Data from Germany, the Czech Republic and the Netherlands indicate that up to 20% of wild boars show positive serum samples for TBEV [27,29,30]. There are most likely two mechanisms of TBEV spread coexisting In the Upper Rhine Valley. We speculate that besides short distance spreading, there are non-continuous distribution patterns. Such long distance imports can explain the close genetic relatedness between the newly identified TBEV strains on both sides of the Rhine river with TBEV strains from eastern Europe. It has been shown that TBEV can be found on ticks sampled from migrating birds and there is evidence that migrating birds are importing TBEV infected ticks from remote eastern areas of Europe, to the very western margins of the current TBEV distribution in ticks and TBE incidence in humans [31].

In conclusion, we demonstrate, to our knowledge for the first time the phylogenetic relations of the newly isolated TBEV strains on both sides of the upper Rhine river. These genetic findings might give us more insights into the dynamics of TBEV spreading and the importance of non-continuous distribution patterns over long distances.

## Supporting information

S1 Table GPS/Glonass data of the sampling sites in France and Germany. (DOCX)

S1 Fig Phylogenetic Data. Phylogenetic analysis of all isolated TBEV samples 2016–2018 using the PhyML algorithm.
(EPS)

S2 Fig Phyleogeography of isolated TBEV (2016–2017) strains in the Upper Rhine Valley. From West to East, Green TBEV strains Robertsau, 2215, HYPR; Red TBEV strains Emmendingen, Aubachstrasse (2016, 2017), HM2, A104; Orange TBEV strains Alsace, Aubachstrasse (2018), Schiltach, 8641, Vlasaty. The marked lines indicate hypothesized corridors/areas of distribution of the specific strains.
(DOCX)
