# Supplementary material for: Phylogenetics of tick-borne encephalitis virus in endemic foci in the upper Rhine region in France and Germany
Source: PLoS One. 2018 Oct 18;13(10):e0204790. doi: 10.1371/journal.pone.0204790 (PMC6193627; doi:10.1371/journal.pone.0204790)
Supplement: S1 Table — (DOCX) [file pone.0204790.s001.docx]

| sampling site | date | GPS/ Glonass data |
| --- | --- | --- |
|  |  |  |
| Burgerwald (G) | May 16 | 48.463594, 7.910760 |
| Foret Neuhof (F) | May 16 | 48.556923, 7.793319 |
| Renchen (G) | May 16 | 48.577751, 7.999355 |
| Aubachstraße (G) | May 16 | 48.639236, 8.123323 |
| Obersasbach (G) | May 16 | 48.637355, 8.113747 |
| Foret de la Robertsau (F) | May 16 | 48.627814, 7.810122 |
| Foret de la Robertsau (F) | May 16 | 48.627638, 7.814857 |
| Sasbachwalden (G) | May 16 | 48.619151, 8.120586 |
| Vogelweg (G) | May 16 | 48.623646, 8.102986 |
| Guebviller (F) | Jun 16 | 47.905083, 7.197065 |
| Schiltach (G) | Mar 17 | 48.294270, 8.320455 |
| Aubachstraße (G) | Mar 17 | 48.639236, 8.123323 |
| Antogast (G) | Mar 17 | 48.475427, 8.181421 |
| Oppenau (G) | Apr 17 | 48.475558, 8.180467 |
| Schiltach (G) | Apr 17 | 48.294270, 8.320455 |
| Sasbachwalden (G) | May 17 | 48.613668, 8.131669 |
| Oppenau (G) | May 17 | 48.475558, 8.180467 |
| Foret de la Robertsau (F) | Jun 17 | 48.620433, 7.816573 |
| Guebviller (F) | Okt 17 | 47.905083, 7.197065 |

S1 Tab GPS/Glonass data of the sampling sites in France (F) and Germany (G)
