# Supplementary material for: Phylogenetics of tick-borne encephalitis virus in endemic foci in the upper Rhine region in France and Germany
Source: PLoS One. 2018 Oct 18;13(10):e0204790. doi: 10.1371/journal.pone.0204790 (PMC6193627; doi:10.1371/journal.pone.0204790)

S2 Fig Phyleogeography of isolated TBEV (2016-2017) strains in the Upper Rhine Valley. From West to East, Green TBEV strains Robertsau, 2215, HYPR; Red TBEV strains Emmendingen, Aubachstrasse (2016, 2017), HM2, A104; Orange TBEV strains Alsace, Aubachstrasse (2018), Schiltach, 8641, Vlasaty. The marked lines indicate hypothesized corridors/areas of distribution of the specific strains.


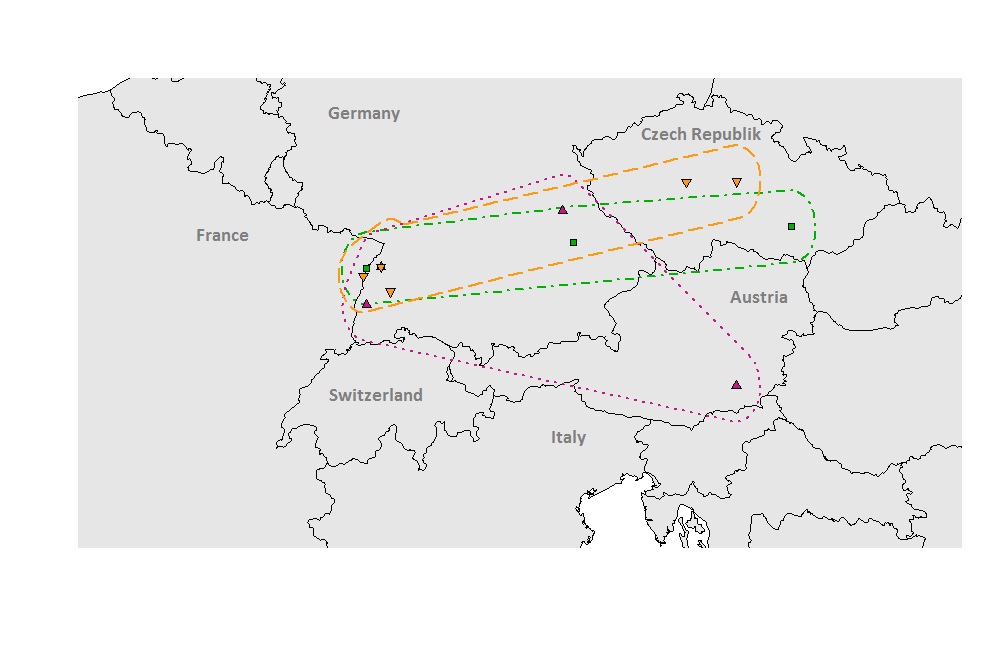

Supplement: S2 Fig — From West to East, Green TBEV strains Robertsau, 2215, HYPR; Red TBEV strains Emmendingen, Aubachstrasse (2016, 2017), HM2, A104; Orange TBEV strains Alsace, Aubachstrasse (2018), Schiltach, 8641, Vlasaty. The marked lines indicate hypothesized corridors/areas of distribution of the specific strains. (DOCX) [file pone.0204790.s003.docx]
